# Regulation of Key Antiplatelet Pathways by Bioactive Compounds with Minimal Bleeding Risk

**DOI:** 10.3390/ijms222212380

**Published:** 2021-11-17

**Authors:** Eduardo Fuentes, Sergio Wehinger, Andrés Trostchansky

**Affiliations:** 1 Thrombosis Research Center, Medical Technology School, Faculty of Health Sciences, Universidad de Talca, Talca 3460000, Chile; 2 Department of Clinical Biochemistry and Immunohematology, Faculty of Health Sciences, Universidad de Talca, Talca 3460000, Chile; 3 Departamento de Bioquímica and Center for Free Radical and Biomedical Research, Facultad de Medicina, Universidad de la República, Montevideo 11800, Uruguay

**Keywords:** bioactive compounds, hemostasis, platelet, thrombosis, bleeding

## Abstract

Cardiovascular disease is strongly influenced by platelet activation. Platelet activation and thrombus formation at atherosclerotic plaque rupture sites is a dynamic process regulated by different signaling networks. Therefore, there are now focused efforts to search for novel bioactive compounds which target receptors and pathways in the platelet activation process while preserving normal hemostatic function. The antiplatelet activity of numerous fruits and vegetables and their multiple mechanisms of action have recently been highlighted. In this review, we review the antiplatelet actions of bioactive compounds via key pathways (protein disulfide isomerase, mitogen-activated protein kinases, mitochondrial function, cyclic adenosine monophosphate, Akt, and shear stress-induced platelet aggregation) with no effects on bleeding time. Therefore, targeting these pathways might lead to the development of effective antiplatelet strategies that do not increase the risk of bleeding.

## 1. Introduction

Cardiovascular disease—a leading cause of morbidity and mortality among adults—is strongly influenced by platelet activation [1]. Platelets are small and specialized disk-shaped cells in the bloodstream released from megakaryocytes and, primarily in hemostasis, can adhere and aggregate at injured vessels to arrest bleeding [2,3]. However, when triggered under pathological conditions, platelet activation leads to thrombotic disorders involved in the pathogenesis of cardiovascular diseases [1,3,4]. Platelet activation and thrombus formation at atherosclerotic plaque rupture sites are dynamic processes regulated by rheological (biomechanical) and soluble-agonist-dependent mechanisms [5], while stabilization of thrombi is supported by the late wave of signaling events promoted by close contact between aggregated platelets [6].

A case-referent study showed a significant reduction of recurrent fatal and non-fatal myocardial infarction with antiplatelet drugs [7]. Currently available antiplatelet agents, including cyclooxygenase 1 inhibitors, P2Y purinoreceptor 12 (P2Y12) antagonists, protease-activated receptor 1 antagonists, and glycoprotein (GP) IIb/IIIa antagonists, inhibit important processes for both thrombosis and hemostasis [8]. Thus, recent clinical studies have shown that the benefit from antiplatelet therapy in primary prevention is counteracted by the entailed bleeding risk [9], and even moderate bleeding was strongly associated with mortality [10].

The antiplatelet activity of numerous fruit and vegetables and their multiple mechanisms of action have recently been highlighted. In this context, mango fruit (*Mangiferaindica*) [11], maqui (*Aristoteliachilensis*) [12], guava (*Psidiumguajava*) [13], tomato pomace [14], cherimoya (*Annonacherimola* Mill.) [15], and lupin (*Lupinus* spp., *Fabaceae* family) [16] have been identified with antiplatelet activity. This activity has been associated with the high content of bioactive compounds like polyphenols, nucleosides, anthocyanins, and carotenoids [11,17,18,19,20]. Of these compounds, guanosine significantly reduced thrombus formation both in vitro and in vivo without significantly affecting bleeding [20].

Bleeding frequently occurs as a serious side effect of antiplatelet drugs due to the disturbance of normal hemostasis [21]. Reducing bleeding complications is one of the primary goals in the study of a novel antiplatelet drug [9,22]. Therefore, the present article aims to highlight the relative contribution of selective targets of antiplatelet bioactive compounds necessary to overcome bleeding.

## 2. Platelet Activation

Platelets are essential in the formation and maintenance of blood and lymphatic vessels [23]. Platelet activation at vascular injury sites involves multiple cell signaling pathways that are coordinated in both time and space and is crucial for hemostasis, but uncontrolled platelet activation leads to pathologic thrombus formation and organ failure [24]. Upon platelet activation, cytoskeleton reorganization is essential for platelet secretion and thrombus formation. Platelets are key contributors to the formation of occlusive thrombi, the major underlying cause of cardiovascular disease. Current antiplatelet drugs that inhibit platelet aggregation are effective in cardiovascular disease treatment. Thus, antiplatelet therapy has reduced the morbidity and mortality associated with thrombotic events; however, the utility of current antiplatelet therapies is limited by the concomitant risk of an adverse bleeding event and is still an issue in vascular diseases [25].

## 3. Antiplatelet Therapy and Bleeding Risk

The risk of bleeding increases in patients on antiplatelet therapy over 75 years of age (mainly aspirin based, prasugrel, and clopidogrel plus aspirin); therefore, this is a critical age where the effectiveness and safety of antiplatelet therapy need to be improved. Bleeding is one of the most critical adverse effects of antithrombotic drugs, and many efforts have been made to discover novel antiplatelet agents without bleeding complications [26,27,28,29,30]. During the past few years, oral and intravenous antiplatelet therapies have been developed with escalating potency to reduce the risk of developing ischemic complications and are a cornerstone of therapy in those with clinical atherothrombotic events [31,32]. Antiplatelet therapy is important in the secondary prophylaxis of adverse cardiovascular events such as myocardial infarction and stroke. The cyclooxygenase inhibitor aspirin remains the most frequently prescribed antiplatelet drug, followed by adenosine diphosphate (ADP) P2Y12 receptor blockers. GPIIb/IIIa antagonists are intravenously available antiplatelet agents preventing platelet-to-platelet aggregation via the fibrinogen receptor. The thrombin receptor inhibitor vorapaxar allows the targeting of yet a third pathway of platelet activation. Despite the advent of novel agents and major advances in antiplatelet treatment over the last decade, atherothrombotic events still impair the prognosis of many patients with cardiovascular disease [33].

Limitations of current therapies include: (i) weak inhibition of platelet function (e.g., aspirin); (ii) blockade of only one pathway of ADP-mediated signaling (e.g., clopidogrel); (iii) slow onset of action (e.g., clopidogrel); (iv) interpatient response variability with poor inhibition of platelet response in some patients (e.g., clopidogrel); (v) inability to convert intravenous into an oral GPIIb/IIIa antagonist therapy; (vi) the inability to completely separate a reduction in thrombotic events from an increase in bleeding events [32]. Studies indicate that an intensification of antiplatelet therapy with prasugrel [34], ticagrelor [35], clopidogrel [10], aspirin [36], clopidogrel plus aspirin [37], vorapaxar [38], apixaban [39], or rivaroxaban [40] is associated with an increased efficacy but often with increased bleeding. The increased bleeding risk may result in drug withdrawal, which possibly exposes patients to serious thrombotic complications [41].

## 4. Bioactive Extracts with Antiplatelet Activity

Several dietary supplements and plant or fruit extracts have been reported to exert beneficial and protective effects over different cardiovascular disease risk factors [42,43,44,45]. These studies are part of a growing area of non-pharmacologic nutraceutical-based treatments for cardiovascular disorders. Among the proposed mechanisms, and of relevance for the current review, is that the supplements and extracts may change hemostasis by modulating arachidonic acid metabolism as well as inhibiting blood platelet activation, i.e., platelet aggregation [46,47].

We are going to discuss well-reported examples on platelet aggregation using tomato pomace and *Syzygium cumini* (L.) Skeels (Myrtaceae) or *Aristoteliachilensis* (Mol.) Stuntz extracts. Tomatoes and tomato products are rich sources of folate, vitamin C, and potassium and contain different phytonutrients, with lycopene as the most prominent carotenoid. In vitro and in vivo studies show that tomato extracts have natural antithrombotic effects [48,49] compatible with the presence of adenosine in the tomato, which inhibits thrombin-induced platelet aggregation [50]. When analyzed, the antiplatelet bioactive compounds present in the extracts involved, apart from adenosine, adenosine monophosphate and guanosine, as well as the adenosine derivatives liguadenosines A and B [51,52]. *Syzygium cumini* (L.) Skeels (*S. cumini*) extract composition has been reported, being the leaf polyphenol-rich extract (PESc) composition determined via HPLC-UV and HPLC-MS/MS, consisting of gallic acid, quercetin, myricetin, and its derivatives myricetin-3-a-arabinopyranoside and myricetin-deoxyhexoside [53]. Moreover, different flavonoids were identified, with myricetin being the most abundant [53]. The polyphenol-rich extract is considered an important source of bioactive compounds against cardiometabolic disorders and its relevance has been reported for many years [54], e.g., its usage in Unani medicine to “enrich blood” [55]. Indeed, hyperactivation of platelets from diabetic patients has been reported using *S. cumini* extracts [56] in addition to the polyphenol-rich extract inhibitory effects on both platelet activation and aggregation. Platelet aggregation induced using the protein kinase C (PKC) activator phorbol 12-myristate 13-acetate (PMA) resulted in it being inhibited by a polyphenol-rich extract, suggesting that some of the bioactive compounds present in the extract were able to cross the platelet cell membrane, probably targeting PKC or downstream molecules, i.e., signaling that occurs at the end of the platelet activation pathway [57]. Similar data has been reported for a green tea flavonoid-rich extract that reduced platelet aggregation and integrin αIIbβ3 activation upon stimulus with ADP, thrombin, or collagen [58]. Among the most active components present in the polyphenol-rich extract are myricetin, gallic acid, and quercetin [57]. Their role in platelet activation and inhibition of aggregation will be individually discussed later.

*Aristoteliachilensis* (Mol.) Stuntz, known as maqui, grows in central and southern Chile and has been used for a long time for medical purposes [59]. Maqui’s most described actions are related to the high content of phenols in its fruit. We have recently identified and quantified a diverse variety of compounds in maqui’s extracts from different variants (Luna Nueva, Morena, and Perla Negra) and different parts of the plant (leaves, immature and mature fruits) [12]. The bioactive compounds found were caffeic and gallic acids, quercetin, rutin, myricetin, catechin, epicatechin, and anthocyanins mainly derived from delphinidin, malvidin, petunidin, cyanidin, and peonidinanthocyanins [12]. In addition to the identification of the compounds, our group evaluated the capacity of extracts from maqui’s variants to modulate platelet aggregation. Maqui extracts decreased platelet aggregation induced by several agonists, in addition to decreasing the exposure of P-selectin and CD63 at the platelet membrane [12]. 

## 5. Compounds That Inhibit Platelet Activation without Affecting Bleeding Time

In this section, we discuss the antiplatelet actions of bioactive compounds via key pathways (protein disulfide isomerase (PDI), mitogen-activated protein kinases (MAPKs), mitochondrial function, cyclic adenosine monophosphate (cAMP), Akt, and shear stress-induced platelet aggregation (SIPA)), and with no effects on bleeding time.

### 5.1. Protein Disulfide Isomerase

Myricetin was tested in both platelet-rich plasma and washed platelets [57]. Platelet aggregation was inhibited in a dose-dependent manner by the flavonoid for either collagen or thrombin receptor-activating peptide-6 (TRAP-6)-induced aggregation. Moreover, fibrinogen binding and alpha-granule secretion induced by the collagen-related peptide is also inhibited by myricetin. All the effects were done at physiologically relevant concentrations [57]. It has been previously reported that myricetin strongly inhibits arachidonic acid-evoked platelet aggregation [60] without affecting cyclooxygenase activity in platelets [60]. We decided to consider PDI, an enzyme that participates in the αIIbβ3 activation necessary for platelet activation and aggregation processes, a potential target for the flavonoid effect [61]. Myricetin, possibly due to non-covalent bonds, can bind to thiol isomerases and inhibits the reductase activity of PDI and endoplasmic reticulum (ER)–resident protein 57 (ERp57). However, preclinical studies demonstrate that deficiency in platelet ERp57 resulted in increased tail bleeding times and delayed thrombus formation [62]. When compared to quercetin, a flavonoid with a similar chemical structure, the observed effects of myricetin on platelet activation were comparable [63]. Quercetin reduces thrombin-induced platelet aggregation as well as platelet activation via specific agonists of PAR1 and PAR4. Meanwhile, quercitrin (Quercetin 3-rhamnoside) significantly inhibited GPVI-mediated platelet signal transduction during cell activation and blocked FeCl_3_-induced arterial thrombus formation in vivo without prolonging bleeding time [64]. 

The ability of myricetin to inhibit platelet aggregation and activation induced by different agonists supports that this flavonoid may act on molecules common to each pathway [57]. Collagen-mediated GPVI platelet activation was discarded as a target for myricetin due to a lack of suppression of platelet spreading to fibrinogen. The effect of myricetin is compatible with the incapacity of platelets from PDI-deficient mice to form proper thrombi on collagen-coated surfaces, even though their platelets spread normally on fibrinogen [65]. Intravital microscopy demonstrates that platelet PDI is important for platelet accumulation but not initial adhesion and fibrin generation following laser-induced arteriolar injury [65]. 

Among 61 plant-derived polyphenolic compounds analyzed from a customized polyphenol library of beverages with reported cardiovascular benefits [66], tannic acid (TA, gallotannin) was identified as the most potent compound capable of binding PDI. The high affinity of TA for PDI is the result of a KD at the low nM level, which is three orders of magnitude lower than that reported for other PDI inhibitors, including quercetin-3-rutinoside, 9 12-O-tetradecanoylphorbol 13-acetate (TPA), or anti-PDI mAb Clone 1D3 [66]. In addition, TA was able to inhibit the binding of PDI to the platelet surface integrin αIIbβ3, a fundamental step for integrin activation and platelet aggregation. In accordance, thrombin-activated platelets exhibited a reduction in the number of platelet membrane-free thiols. When analyzing platelet aggregation in washed platelets, apart from inhibiting PDI, TA affects G protein-coupled receptors and immunoreceptor tyrosine-based activation motif platelet pathways without any platelet toxicity by TA. In mice, treatment with TA did not exert an increase in the mouse jugular vein and tail bleeding time, thus inhibiting thrombus formation without affecting hemostasis. Overall, TA is a natural inhibitor of PDI with antithrombotic potency without affecting physiological hemostasis [66]. Similarly, juglone from *Juglandaceae* plants inhibited in vitro platelet activation via inhibition of Akt activation and PDI activity [67].

### 5.2. Mitogen-Activated Protein Kinases

Interestingly, MAPKs expressed in platelets, such as ERK, JNK, and P38MAPK, have different roles during platelet activation. On the one hand, agonist-induced MAPKs activation plays a role in platelet secretion, and on the other, integrin-mediated MAPKs activation is important in facilitating clot retraction [68,69]. Dihydromyricetin (DHM) is a flavonol compound found in many dietary foods and plants with established beneficial activities on the metabolic systems [70]. Dihydromyricetin has been reported to be the most abundant flavonoid in Ampelopsis grossedentata, presenting important antithrombotic effects [71]. In a laser injury-induced thrombosis model, DHM treatment was able to decrease both platelet accumulation and fibrin generation; importantly, the former effects were observed without prolonging ex vivo plasma coagulation or tail bleeding time [70]. The inhibition of MAPKs activation, decreasing ERK1/2 and p38 phosphorylation, and suppression of calcium mobilization in platelets support the antithrombotic effects of DHM [70]. Similarly, ginsenoside-Rp3 (*Panax ginseng*), an antiplatelet and antithrombotic compound, significantly attenuated the phosphorylation of the MAPKs molecules ERK and JNK in a dose-dependent manner [69].

### 5.3. Mitochondrial Function

3-methyl quercetin is a methylated flavonol present in leaves, flowers, and the fruit of many plants, also called isorhamnetin [17]. Isorhamnetin corresponds to the 3′-O-methylated metabolite of quercetin, present mainly as glycosides in citrus fruit juices such as sweet orange, tangerine, lemon, and grapefruit. Most of the anti-inflammatory and skin protective actions of isorhamnetin are ascribed to its glycosylated derivatives, but studies in washed platelets have shown that isorhamnetin exerts a significant inhibition on collagen- and TRAP-6-induced platelet aggregation, with half-maximum inhibitory concentration (IC_50_) values in the micromolar order [17]. When PKC was directly activated by PMA in the presence of isorhamnetin, platelet aggregation did not change, suggesting that isorhamnetin acts upstream of PKC. A proposed mechanism of action involves the capacity of isorhamnetin to affect mitochondrial function [72,73]. A reduction of adenosine triphosphate (ATP) levels in platelets was observed in the presence of isorhamnetin. The inhibition was not due to platelet cytotoxicity and the mitochondrial apoptotic-like event phosphatidylserine exposure was also not affected [17]. Inhibition of mitochondrial function is also supported by the observed increase in intraplatelet calcium levels [72,73]. Isorhamnetin, in addition to the changes in ATP levels, decreased platelet mitochondrial membrane potential without affecting reactive oxygen species (ROS) production [17].

Other studies have shown that xanthohumol prevents both venous and arterial thrombosis without incurring a bleeding risk. Xanthohumol is a prenylated flavonoid that is present in several flowers, e.g., Hops (*Humuluslupulus* L.) used for beer preparation, and it is present in the the prenyl group bounded to ring A [74,75]. Xanthohumol has been reported to exert protective effects in metabolic syndrome and related diseases [74]. The mechanism involves inhibition of platelet activation via induction of Sirt1 expression, decreasing ROS accumulation, and inhibiting platelet mitochondrial DNA (mtDNA) release [76].

Mitochondrial pyruvate dehydrogenase kinases (PDK 1–4) play a pivotal role in metabolic flexibility by inhibiting the pyruvate dehydrogenase complex. Human and mouse platelets pretreated with dichloroacetic acid (DCA), a potent inhibitor of all four forms of PDK, exhibited decreased platelet aggregation and exhibited decreased aerobic glycolysis in response to convulxin only. Wild-type mice pretreated with DCA were less susceptible to thrombosis without altering hemostasis [77,78].

### 5.4. Cyclic Adenosine Monophosphate

The endogenous nucleoside and natural product guanosine has intercellular signaling capacity with anti-inflammatory and neuroprotective effects, and guanosine concentrations increase during specific pathological conditions such as hypoxia and/or hypoglycemia [79,80]. A few years ago, our group demonstrated that guanosine exerts antiplatelet and antithrombotic activities, in a dose-dependent manner, being the mechanism of action mediated by adenosine [20]. Guanosine diminishes ADP-induced platelet aggregation and limits thrombotic risk. Guanosine antiplatelet effects were associated with the activation of the cAMP/protein kinase A (PKA) signaling pathway and a reduction in thromboxane B2 levels. Importantly, guanosine, without affecting bleeding, reduces thrombus formation both in vitro and in vivo [20], while Ginsenoside-Rp1 (*Panax ginseng*) elevated cAMP levels and increased vasodilator-stimulated phosphoprotein (VASP) ser239 and inhibited in vivo thrombus formation and ex vivo platelet aggregation and ATP secretion without affecting tail bleeding time and coagulation time, respectively [81]. Similarly, an antiplatelet mechanism with elevated cAMP levels has been described in Ginsenoside-Rp3 [69]. Antiplatelet activity of caffeic acid on platelet-mediated thrombosis in vivo, which is at least partly mediated by interference in phosphorylation of ERK, p38, and JNK, leads to elevation of cAMP. In addition, caffeic acid significantly inhibited thrombus formation in vivo and did not significantly prolong the tail bleeding time in mice either [82]. 

### 5.5. Akt Pathway

Psm2, one of the pyrrolidinoindoline alkaloids isolated from whole *Selaginella moellendorffii* plants, has been shown to present potent antiplatelet activity. Psm2 dose-dependently inhibited human platelet aggregation, decreasing the thrombus formation via inhibition of the phosphatidylinositol 3-kinase (PI3K)/Akt pathway, and produced only slight bleeding in a mouse tail cutting model [83]. Similarly, tripeptide SQL (H-Ser-Gln-Leu-OH), via blocking PI3K-mediated signaling, inhibited platelet aggregation and thrombus formation in vivo, without increasing the bleeding time in mice [84]. Gintonin is a non-saponin bioactive component of ginseng that remarkably inhibited collagen pathway (SFK, Syk, phospholipase C (PLC) γ2, MAPK, and PI3K/Akt)-induced platelet aggregation and suppressed thrombus formation with modestly extended bleeding [85]. 6,7-dihydroxycoumarin, also known as esculetin, is the main active ingredient of the traditional Chinese medicine *Cortex Fraxini* [86]. Esculetin inhibits human platelet activation by hindering the PLCγ2-PKC cascade, hydroxyl radical formation, and Akt activation. Furthermore, esculetin significantly increased the occlusion time in thrombotic platelet plug formation and did not prolong the bleeding time [87]. Sulforaphane, a dietary isothiocyanate found in cruciferous vegetables, prevented PI3K/Akt signaling, prevented platelet aggregation, and reduced thrombus formation inflow conditions [88]. Additionally, sulforaphane possesses antiplatelet activity via activation of adenylate cyclase/cAMP [89]. Neferine is a bis-benzylisoquinoline alkaloid that inhibits platelet activation via blocking of PI3K activation and decreases the levels of phosphorylation of Akt, GSK3β, and p38 MAPK in platelets [90]. Neferine can significantly reduce the area of mice platelet adhesion to the collagen and inhibits thrombosis in vitro. In a collagen-epinephrine-induced acute pulmonary thrombus mouse model, neferine, at 6 mg/kg, significantly attenuated thrombus formation [91]. 

Licochalcone A, a major phenolic chalcone constituent of the licorice species Glycyrrhiza inflata, has been reported to have anti-inflammatory effects, especially when using topically [92]. Licochalcone A effectively reduced platelet activation and thrombus formation, without inducing bleeding, in part through the inhibition of PLCγ2-PKC, Akt, and MAPK pathways [93]. Morin Hydrate (3,5,7,2′,4′-pentahydroxyflavone) is a polyphenol compound that has been extensively studied for different pharmacological activities in various human disorders, with slight side effects. Morin hydrate crucially inhibits platelet activation through inhibition of the PLCγ2-PKC cascade and subsequent suppression of Akt and MAPK activation. Moreover, morin hydrate substantially increased the occlusion time of thrombotic platelet plug formation but did not affect the bleeding time in mice [94].

### 5.6. Shear Stress-Induced Platelet Aggregation

SIPA, which occurs under abnormally high shear stress, plays a crucial role in the development of arterial thrombotic diseases. Of note, SIPA is a promising target to overcome bleeding since SIPA happens only under pathological conditions. In isolated human platelets, protocatechuic acid (PCA) decreased SIPA. Antithrombotic effects of PCA were confirmed in vivo in a rat arterial thrombosis model, where PCA significantly delayed the arterial occlusion induced by FeCl_3_. Of note, PCA did not increase bleeding times in a rat tail transection model [95]. The effects of paeoniflorin showed inhibition of SIPA and significantly prevented arterial thrombosis in vivo without prolonging bleeding time or blood clotting time in rats [96]. Cyanidin-3-glucoside inhibits human platelet activation, aggregation, and secretion and downregulates the collagen-GPVI signaling pathway and thrombus formation (both venous and arterial shear stresses) without prolonging the bleeding time in mice [97]. Delphinidin-3-glucoside reduced thrombus growth in human and murine blood in perfusion chambers at both low and high shear rates, and no significant difference in tail bleeding times was observed [98]. The antiplatelet action of tetramethylpyrazine was selective by inhibiting the platelet thrombus formation under high shear rates [99]. 

Thrombosis, chronic inflammation, and fibrosis are at the end of the pathological interactions of activated endothelium, neutrophils, and platelets [100]. Either pure or food-derived polyphenols have been reported to decrease endothelial dysfunction and endothelial cell activation in vitro, ex vivo, and in animal models of endothelial dysfunction by decreasing oxidant production. Therefore, polyphenols reduce the interaction of platelets with activated endothelial cells by increasing the availability of nitric oxide, thus preventing platelet aggregation [101].

The effect of each bioactive compound on bleeding time is described in Table 1. 

**Figure 1:**
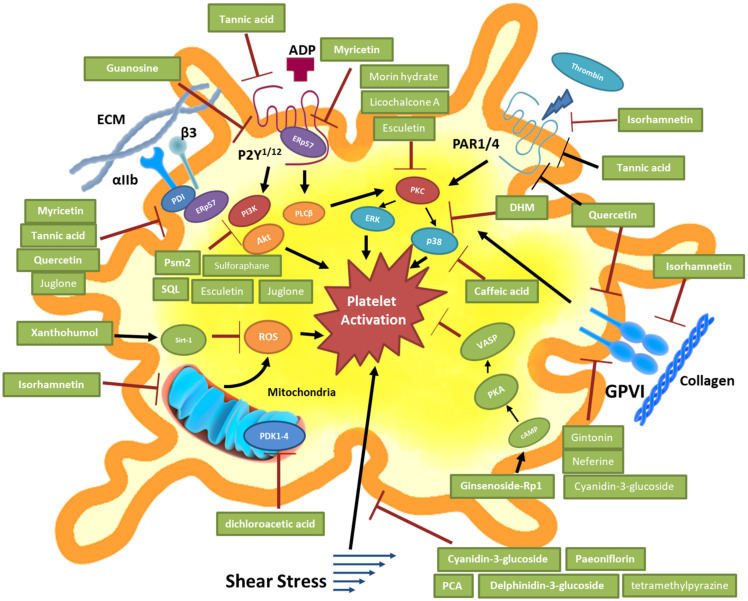
Antiplatelet targets of bioactive compounds without bleeding risk. In red lines: inhibition, black arrows: activation. DHM: dihydromyricetin, PCA: protocatechuic acid. SQL: tripeptide H-Ser-Gln-Leu-OH.

The examples presented above exert their antiplatelet activities through the additive, cooperative, or synergic action of the bioactive compounds present in plants’ or fruits’ extracts (Figure 1). 

## 6. Potential and Pitfalls of the Therapeutic Use of Antiplatelet Bioactive Compounds

Most of the data presented above were obtained from observational studies using platelet-rich plasma, washed platelets, or blood samples in vitro or using mice models [102]. In addition, the bioactive compounds were obtained commercially or present in aqueous, hydroalcoholic, or ethanolic extracts from different plant leaves or fruits. Thus, implementations of clinical trials with either the pure compounds or the extracts are necessary to the development of novel, natural antithrombotic drugs.

An important issue to be evaluated for the use of the extracts from plants or fruit is the type of solvents used to obtain the mixture of bioactive compounds, i.e., methanol, ethanol, and hydroalcoholic mixtures. In addition, it is relevant to perform the correct and precise determination for both composition and quantities of the compounds to avoid toxicity nor non-desired side effects. Most of the available clinical trials use foods, mainly from berries, cocoa, or chocolate, and less frequently extracts from berries and green tea [102]. It is important to point out the lack of trials using the type of extracts presented before as an important pitfall of the use of these nutraceutical extracts with antiplatelet or antithrombotic potential. Moreover, half of the trials performed in the last 20 years were done on healthy volunteers, while less than 20% involve people with at least one cardiometabolic risk factor. From the total number of trials with polyphenols in the last 20 years, although 20% analyzed vascular and endothelium responses, there is a lack of trials on platelet function and thrombosis [102].

Finally, an additional relevant fact for the bioactive effects of the referenced compounds is their pharmacokinetics, absorption with chemical modifications suffered by the polyphenols during the process, as well as their transport to platelets to exert their effects [103]. The latter is relevant for the interaction with other antiplatelet drugs. One example was a synergy on anti-aggregation effects when dietary flavonoids and their metabolites were administered with aspirin [104]. Thus, it may be suggested that the co-administration of dietary polyphenols in conjunction with antiplatelet drugs may enhance therapeutic effects. However, it should not be the case. Polyphenols undergo liver and intestinal biotransformation during metabolism, while they can also suppress cytochrome P450 enzyme activity found in both organ sites [105,106]. Cytochrome P450 enzymes are involved in drug metabolism; thus, modification of their activity may increase unfavorable drug circulating levels. Hence, although polyphenols may possess antiplatelet properties their coadministration may not be safe. Overall, in vivo and trial studies evaluating possible polyphenol-drug interactions are necessary to address these issues. 

## 7. Conclusions

The development of novel antiplatelet and antithrombotic drugs is an area of study with increased visibility. The sources of these compounds, e.g., naturally or chemically synthesized, as well as the mechanisms of action are important facts to develop new studies, clinical trials, and their use in human patients. Moreover, their capacity to decrease platelet aggregation and thrombus formation without changing bleeding time is a challenge when developing antiplatelet drugs. Due to extensive studies on pharmacokinetics and toxicity in animal and humans studies, quercetin, myricetin, and some anthocyanins seem to be the compounds of choice to perform clinical studies to determine their potential to develop naturally derived antiplatelet drugs. This review gives an extensive discussion on the different compounds, mechanisms of action, and desired and undesired side effects to aid researchers in the design of studies in the cardiovascular disease area.

## Figures and Tables

**Table 1:** Antiplatelet compounds without increasing bleeding time.

Compound	Natural Sources *	Effects and Proposed Mechanisms	In Vitro or In Vivo Effects	Concentration Ranges In Vitro	Effects on Bleeding	Reference
Myricetin (from *Syzygium cumini* leaf)	*Syzygium cumini* (L.)	- Inhibition of aggregation induced by collagen or TRAP-6 - Inhibition of fibrinogen binding and alpha-granule secretion induced by CRP - The mechanism involves PDI inhibition	In vitro (human platelet-rich plasma and washed platelet) In vivo (*Mus musculus*)	10–100 µM	No changes in bleeding time were observed in mice supplemented with myricetin	[57]
Quercitrin (comercial product)	As quercitrin (3-rhamnoside) in many fruit and vegetables: apples, honey, raspberries, onions, red grapes, cherries, citrus fruits	- Impaired CRP-induced platelet aggregation, granule secretion, reactive oxygen species generation, and intracellular calcium mobilization - Inhibited outside-in signaling of αIIbβ3 integrin - Inhibition of the GPVI-mediated platelet activation	In vivo *(Mus musculus)* In vitro (thrombus formation on collagen-coated surfaces under arteriolar shear)	10–30 µM	Without prolonging bleeding time	[64]
Tannic acid (comercial product)	Aerial plant tissues, gall nuts	- Inhibition of platelet aggregation stimulated by either GPVI or ITAM pathway agonists - Reduction of platelet adhesion and spreading on immobilized fibrinogen - High affinity for PDI, whose inhibition explains the mechanism of action	In vitro (isolated human platelets) In vivo (*Mus musculus*)	1–100 µM	The administration did not change mouse jugular vein and tail bleeding time	[66]
Juglone (comercial product)	Roots, leaves, and hulls of Juglandaceae plants	- Inhibited platelet aggregation and αIIbβ3 activation - Reduced thrombus formation in vitro - Abolished intracellular Ca^2+^ elevation and protein kinase C activation - Inhibition of Akt and PDI	In vitro (collagen-coated flow chambers)	1–5 µM	Nd.	[67]
Dihydromyricetin (Comercial product)	The most abundant flavonoid in *Ampelopsis grossedentata*	- Modulation of platelet aggregation, secretion, adhesion, spreading, and integrin activation - Inhibition of phosphatidylserine exposure - The mechanism involves attenuation of calcium mobilization and ERK1/2 and p38 phosphorylation	In vitro (washed platelets and HUVEC cells) In vivo (C57BL/6j mice) Ex vivo (plasma coagulation)	50–200 μg/mL	Supplementation did not affect prolonging ex vivo plasma coagulation or tail bleeding time	[70]
Ginsenoside-Rp3 (reduced version of ginsenoside-Re from *Panax ginseng* C.A. Mayer)	*Panax ginseng* C.A. Mayer	- Inhibited platelet aggregation - Suppressed Ca^2+^ mobilization, ATP release, and P-selectin expression, integrin αIIbβ3, fibronectin adhesion, and clot retraction - The mechanism involves elevated cAMP levels and VASP phosphorylation and attenuated MAPKs, Src, PLCγ2, PI3K/Akt, and Src family kinases (Src, Fyn, and Lyn) - Protected mice from thrombosis	In vitro (platelets of human and SD rats) In vivo (C57BL/6 J male mice)	6.25–50 µM	Nd.	[69]
Isorhamnetin (Comercial product)	*Phaseolus vulgaris* L. and leaves, flowers, and fruits of many plants (pears, olive oil, wine, tomato, parsley, green bell peppers, and dills)	- Inhibition of collagen and TRAP-6 induced platelet aggregation with IC_50_ in the micromolar order - Decrease of ATP levels - The mechanism involves affection of mitochondrial function	In vitro (washed platelets)	10–100 μM	Nd.	[17]
Xanthohumol (from *Humulus lupulus* cones)	*Humuluslupulus* cones	- Inhibition of platelet activation - Inhibition of mtDNA release - Decrease of ROS overload - Induction Sirt1 expression	In vivo (C57/BL6 mice, rats, and Sprague-Dawley) In vitro (washed platelets from rats)	0.05–5 µM	Inhibited carotid arterial and inferior vena cava thrombosis without a significant risk of bleeding in mice	[76]
Dichloroacetic acid (comercial product)	*Asparagopsistaxiformis*	- Inhibition of pyruvate dehydrogenase kinase - Decreased aerobic glycolysis - Inhibited ATP secretion, TXA2 generation, and tyrosine phosphorylation of Syk and PLCγ2 in the GPVI signaling pathway	In vitro (human and mouse washed platelets) In vivo (C57/BL6 mice)	10–25 mM	Less susceptible to thrombosis in the FeCl_3_-induced carotid and laser injury-induced mesenteric artery thrombosis without altering hemostasis in mice	[77,78]
Guanosine (comercial product)	Pancreas, clover, coffee beans, pollen from pines, sugar beets, yeast, and fish scales	- Marked inhibition of platelet activation stimulated by ADP - Mechanisms propose the activation of the cAMP/PKA signaling pathway - Effects on platelets are mediated by adenosine	In vitro (human washed platelets) In vivo (C57BL/6 mice)	10–500 μmol/L	Significant reduction of thrombus formation both in vitro and in vivo without significantly affecting bleeding	[20]
Ginsenoside-Rp1 (comercial product)	*Panax ginseng* C.A. Mayer	- Inhibited platelet aggregation - Elevated cAMP levels and increase in phospho-VASPser239 - Suppressed collagen-induced ATP-release, thromboxane secretion, p-selectin expression, Ca^2+^ mobilization, and αIIbβ3 activation and attenuated p38(MAPK) and ERK2 activation - Inhibited tyrosine phosphorylation of multiple components (Fyn, Lyn, Syk, LAT, PI3K, and PLCγ2) of the GPVI signaling pathway	In vitro (washed platelets from rats) In vivo (Sprague–Dawley rats and C57BL/6J mice)	2.5–20 μg/L	Without affecting tail bleeding time and coagulation time	[81]
Caffeic acid (comercial product)	In many fruit and vegetables: *Salvia* miltiorrhizae, olives, coffee beans, fruits, potatoes, carrots, and propolis	- Inhibited thrombus formation in vivo - Inhibited ADP-induced platelet aggregation, P-selectin expression, ATP release, Ca^2+^ mobilization, and integrin αIIbβ3 activation - Attenuated p38, ERK, and JNK activation and enhanced cAMP levels	In vitro (mouse platelets) In vivo (C57BL/6J mice)	25–100 μM	Did not significantly prolong the tail bleeding time in mice either	[82]
Psm2 (from *Selaginella moellendorffii*)	*Selaginella moellendorffii*	- Inhibited human platelet aggregation in vitro/ex vivo - Decreased the thrombus formation - Did not affect the binding of fibrinogen to αIIbβ3 - Inhibited PI3K/Akt	In vivo (Sprague-Dawley and ICR mice) In vitro (EA.hy926 cells) Ex vivo (human and rat platelets)	0.3–3 mg/mL	Produced only slight bleeding in a mouse tail cutting model	[83]
Tripeptide SQL (H-Ser-Gln-Leu-OH) (synthesized by the author’s laboratory)	*Scolopendra subspinipes* mutilans	- Inhibited platelet aggregation - Attenuated thrombus formation - Binds PI3Kβ - Inhibition of Akt Ser473 phosphorylation	In vivo (ICR mice, Sprague Dawley rats, and New Zealand white rabbits) In vitro (human platelets)	0.27 mg/mL	Did not prolong the bleeding time in mice	[84]
Gintonin (from *Panax ginseng*)	*Panax ginseng*	- Impairment in GPVI signaling molecules, including SFK, Syk, PLCγ2, MAPK, and PI3K/Akt	In vitro (washed Sprague-Dawley rat platelets) In vivo (C57BL/6J mice)	12.5–50 μg/mL	Modestly extended bleeding	[85]
Esculetin (comercial product)	*Artemisia capillaries*, *Cortex fraxini.*	- Inhibited collagen- and arachidonic acid-induced platelet aggregation - Inhibited adenosine triphosphate release, P-selectin expression, OH· formation, Akt activation, and PLCγ2/PKC phosphorylation - Increased the occlusion time in thrombotic platelet plug formation	In vitro (human platelets) In vivo (ICR mice)	10–80 μM	Did not prolong the bleeding time	[87]
Sulforaphane (comercial product)	Cruciferous vegetables: *Brassica oleracea* var. italic, var. gemmifera, and var. Capitata.	- Inhibited human platelet aggregation - Reduced thrombus formation in vitro - Inhibited the PI3K/Akt pathway	In vitro (washed human platelets)	10–200 μM	Nd.	[88]
Morin hydrate (comercial product)	*Chlorophora tinctoria, Maclura pomifera, Prunus dulcis, Chlorophora tinctoria*, onion, apple, and other Moraceae.	- Inhibited platelet aggregation stimulated by collagen - Inhibited ATP release, intracellular Ca^2+^ mobilization, P-selectin expression, and phosphorylation of PLCγ2, PKC, and Akt - Diminished ERK2 or JNK1 activation, except for p38 MAPK - Increased the occlusion time of thrombotic platelet plug formation	In vitro (washed human platelets) In vitro (ICR mice)	20–80 μM	Did not affect bleeding time in mice	[94]
Neferine (from *Nelumbo nucifera* Gaertn)	*Nelumbo nucifera*.	- Suppressed platelet aggregation - Inhibited thrombin-induced platelet P-selectin expression, PAC-1, and fibrinogen binding - Reduced the adhesion of human platelets on coated collagen - Inhibited the spreading of human platelets on immobilized fibrinogen - Inhibited the PI3K-Akt-GSK3β/p38 MAPK signaling pathway - Inhibited thrombosis in vitro	In vitro (washed human and Kunming mouse) platelet) Ex vivo (Kunming mice	0.3–3 μM	Nd.	[90,91]
Licochalcone A (comercial product)	*Glycyrrhiza spp.*	- Inhibited platelet aggregation induced by collagen - Markedly attenuated collagen-stimulated ATP release, P-selectin secretion, calcium mobilization, and αIIbβ3 activation - Reduced the activation of PLCγ2, PKC, Akt, and MAPKs - Prevented ADP-induced acute pulmonary thrombosis	In vitro (human washed platelets In vivo (ICR and C57BL/6 mice)	2–10 μM	Did not increase bleeding times	[93]
Protocatechuic acid (comercial product)	*Lonicera* flowers, *Oryza sativa* L., and *Allium cepa* L.	- Decreased SIPA - Inhibited platelet activation, including intracellular calcium mobilization, granule secretion, and adhesion receptor expression - Antithrombotic effects	In vitro (isolated human platelets) In vivo (Sprague Dawley rats) Ex vivo (Sprague Dawley rats)	1–25 μM	Did not increase bleeding times	[95]
Paeoniflorin (comercial product)	*Paeonia suffruticosa*.	- Highly selective against SIPA, through modulating vWF-platelet GPIb interaction - Prevented arterial thrombosis in vivo	In vitro (washed human platelets In vivo (Sprague-Dawley rats) Ex vivo (rats-derived PRP)	10–250 μM	Without prolonging bleeding time or blood clotting time in rats	[96]
Cyanidin-3-glucoside (comercial product)	Fruits and vegetables: mulberries, grapes, blackberries, and red cabbage.	- Inhibited (*p* < 0.05) human platelet adhesion and aggregation to collagen at both venous and arterial shear stresses - Inhibited platelet activation, secretion, fibrinogen binding, and aggregation - Downregulated collagen-induced GPVI signaling - Attenuated thrombus growth	In vitro (washed human platelets) In vivo (C57BL/6J mice)	0.5–50 μM	Without prolonging bleeding time in mice	[97]
Delphinidin-3-glucoside (comercial product)	Fruit and vegetables: mulberries, grapes, blackberries, and red cabbage.	- Inhibited human and murine platelet aggregation - Reduced thrombus growth - Inhibited the expression of P-selectin, CD63, CD40L, which reflect platelet α- and δ-granule release, and cytosol protein secretion - Reduced phosphorylation of adenosine monophosphate-activated protein kinase	In vitro (gell-filtered human and murine platelets) In vivo (C57BL/6J mice)	0.5–50 μM	Did not significantly affect bleeding time in mice	[98]
Tetramethylpyrazine (comercial product)	*Ligusticum chuanxiong,* cacao beans, soybeans.	- Inhibits shear-induced platelet aggregation under relatively high shear rate - Inhibited P-selectin surface expression and microparticle release	In vitro (PRP from humans)	0.9–3.7 mM	Bleeding was not determined, but no significant influences were observed under relatively low shear rates	[99]

* Natural sources independent of the study described. Nd.: not determined. ADP: adenosine diphosphate, ADP: adenosine diphosphate, ATP: adenosine triphosphate, cAMP: cyclic adenosine monophosphate, CRP: collagen-related peptide, GP: glycoprotein, HUVEC: human umbilical vein endothelial cells, ITAM: immunoreceptor tyrosine-based activation motif, MAPKs: mMitogen-activated protein kinases, mtDNA: mitochondrial DNA, OH•: hydroxyl radical, PDI: protein disulfide isomerase, PKA: protein kinase A, PKC: protein kinase C, PLC: phospholipase C, PRP: pPlatelet-rich plasma, ROS: reactive oxygen species, SIPA: shear stress-induced platelet aggregation, TRAP-6: thrombin receptor-activating peptide-6, TXA2: thromboxane A2, VASP: vasodilator-stimulated phosphoprotein, vWF: Von Willebrand factor.
